#  Hesitação vacinal infantil e COVID-19: uma análise a partir da percepção dos profissionais de saúde 

**DOI:** 10.1590/0102-311XPT061523

**Published:** 2024-03-11

**Authors:** Ester Paiva Souto, Michelle Vieira Fernandez, Celita Almeida Rosário, Priscila Cardia Petra, Gustavo Correa Matta

**Affiliations:** 1 Escola Nacional de Saúde Pública Sergio Arouca, Fundação Oswaldo Cruz, Rio de Janeiro, Brasil.; 2 Universidade de Brasília, Brasília, Brasil.; 3 Instituto Aggeu Magalhães, Fundação Oswaldo Cruz, Recife, Brasil.

**Keywords:** Hesitação Vacinal, Pandemias, COVID-19, Programas de Imunização, Políticas Públicas de Saúde, Vaccination Hesitancy, Pandemics, COVID-19, Immunization Programs, Public Health Policies, Vacilación a la Vacunación, Pandemias, COVID-19, Programas de Inmunización, Políticas Públicas de Salud

## Abstract

Este artigo apresenta os resultados de uma pesquisa sobre a percepção dos profissionais de saúde sobre a hesitação vacinal infantil relacionada à COVID-19. Baseado no constructo teórico da hesitação vacinal, foi realizada uma pesquisa qualitativa com 86 trabalhadores da atenção primária à saúde (APS) em quatro municípios de quatro estados brasileiros e no Distrito Federal. A análise temática foi realizada e obtiveram-se três categorias: medo, desinformação em vacina e papel dos profissionais de saúde. O medo como motivo de hesitação vacinal gerou reflexões sobre a condução da pandemia pelo Governo Federal, principalmente no que tange à governabilidade por meio desse afeto, e sobre as consequências do uso das plataformas digitais na população. O medo relacionou-se ao fato de a vacina ainda ser percebida como experimental; às possíveis reações adversas; à ausência de estudos de longo prazo; à falsa percepção de risco reduzido da COVID-19 em crianças; e às condutas do Governo Federal geradoras de insegurança nos efeitos da vacina. A desinformação em vacina relacionou-se às *fake news* sobre a vacina e suas reações; ao fenômeno da infodemia e desinformação; e à ausência de orientação e conhecimento sobre vacinas. Por fim, o trabalho discute o papel fundamental dos profissionais de saúde da APS no aumento da cobertura vacinal devido à confiabilidade perante a população e à proximidade com os territórios, fatores que possibilitam reverter o medo e a desinformação diante das vacinas. Ao longo do trabalho, buscou-se apresentar as convergências entre o conteúdo dos temas delineados e os determinantes da hesitação vacinal e refletir sobre possibilidades para a reconstrução da alta adesão às vacinas infantis.

## Abstract (en)

This article presents the results of a study on health professionals’ perceptions of childhood vaccine hesitancy related to COVID-19. Based on the theoretical construct of vaccine hesitancy, a qualitative study was conducted with 86 primary health care (PHC) workers in four municipalities in four Brazilian states and in the Federal District. A thematic analysis was performed and three categories were obtained: fear, misinformation about vaccines, and the role of health professionals. Fear as a reason for vaccine hesitancy has led to reflections on the Brazilian Federal Government’s management of the pandemic, especially regarding governability and the consequences of the use of digital platforms on the population. Fear was related to the vaccine still being perceived as experimental; to the adverse reactions it may cause; to the lack of long-term studies; to the false perception of reduced risk of COVID-19 in children; and to the Federal Government’s behavior, which creates uncertainty about the effects of the vaccine. Vaccine misinformation was related to fake news about the vaccine and its reactions; the phenomenon of infodemic and misinformation; and the lack of guidance and knowledge about vaccines. Finally, the article discusses the fundamental role of PHC workers in increasing vaccination coverage due to the trust among the population and proximity to the territories, factors that enable the reversal of fear and misinformation about vaccines. Throughout the study, authors’ sought to show the convergences between the content of the themes outlined and the determinants of vaccine hesitancy and to consider possibilities for rebuilding high adherence to childhood vaccines.

## Resumen

Este artículo presenta los resultados de una encuesta sobre la percepción de los profesionales de la salud acerca de la reticencia vacunal infantil relacionada con la COVID-19. Con base en el constructo teórico de la reticencia vacunal, se realizó una encuesta cualitativa con 86 trabajadores de la atención primaria de salud (APS) en 4 municipios de 4 estados brasileños y en el Distrito Federal. Se realizó un análisis temático y se obtuvieron tres categorías: miedo, desinformación sobre vacunas y papel de los profesionales de la salud. El miedo como motivo de reticencia vacunal dio lugar a reflexiones sobre el manejo de la pandemia por parte del Gobierno Federal, sobre todo en lo que respecta a la gobernabilidad por medio de esta afección y las consecuencias del uso de plataformas digitales en la población. El temor se relacionó con el hecho de que la vacuna todavía se percibe como experimental; con las reacciones adversas que puedan provocar; con la ausencia de estudios a largo plazo; con la falsa percepción de riesgo reducido de COVID-19 en niños y con las conductas del Gobierno Federal que generan inseguridad sobre los efectos de la vacuna. La desinformación sobre las vacunas se relacionó con noticias falsas sobre la vacuna y sus reacciones; el fenómeno de la infodemia y la desinformación; y la ausencia de orientación y conocimiento sobre las vacunas. Finalmente, el trabajo discute el papel fundamental de los profesionales de la salud de la APS en el aumento de la cobertura vacunal debido a su confiabilidad entre la población y cercanía a los territorios, factores que permiten revertir el miedo y la desinformación respecto a las vacunas. A lo largo del trabajo, se buscó presentar las convergencias entre el contenido de los temas delineados y los determinantes de la reticencia vacunal y reflexionar sobre las posibilidades para la reconstrucción de una alta adhesión a las vacunas infantiles.

## Introdução

No contexto da pandemia de COVID-19, a vacinação foi uma estratégia desenvolvida com êxito para a proteção da população e para o enfrentamento coletivo da emergência sanitária. Os primeiros esforços no desenvolvimento das vacinas iniciaram-se em março de 2020 e, ao longo desse ano, laboratórios como Pfizer e Sinovac divulgaram as etapas dos seus ensaios clínicos, processos acompanhados por todos com atenção ^1^. Com a aprovação da Agência Nacional de Vigilância Sanitária (Anvisa), os primeiros imunizantes chegaram ao Brasil e a vacinação se iniciou, em janeiro de 2021, nos profissionais de saúde e idosos ^2^.

O primeiro ano da pandemia foi marcado pela falta de coordenação do Governo Federal, levando estados e municípios a adotarem ações díspares para o enfrentamento da emergência sanitária ^3^, conjuntura replicada na implementação da vacinação contra a COVID-19 ^4^. Após um ano sem alinhamento entre os entes federativos, acrescido da grande circulação de notícias falsas ^5^, no início de 2022, o Ministério da Saúde ainda insistia na eficácia do tratamento precoce, contrariamente aos dados científicos já disponíveis ^6^.

No caso da imunização das crianças, o cenário foi ainda mais complexo. Em junho de 2021, a Anvisa autorizou a aplicação do imunizante da Pfizer na população acima dos 12 anos. Em dezembro do mesmo ano, a agência aprovou a vacina para crianças a partir dos cinco anos ^7^. No entanto, o Ministério da Saúde relutou em iniciar a imunização dessa faixa etária. Em janeiro de 2022, enquanto 79% da população apoiava a vacinação das crianças entre cinco e 12 anos ^8^, o ex-presidente Jair Bolsonaro se manifestou contra o imunizante, afirmando que o número de crianças mortas pela COVID-19 era insignificante ^9^. A realidade, porém, demonstrava o oposto: a COVID-19 foi letal em 7,6% das 3.138 crianças e adolescentes internadas até o final de 2021 ^10^.

Determinantes relativos à descoordenação do Governo Federal e às incertezas em torno da vacina estão relacionados à decisão em aderir ou não à vacinação. Nesse desígnio, em 2012 a Organização Mundial da Saúde (OMS), por meio do grupo de trabalho em hesitação vacinal SAGE (*Strategic Advisory Group of Experts on Immunization*), propôs o conceito de hesitação vacinal como o atraso na aceitação ou recusa da vacinação, apesar da disponibilidade nos serviços ^11^. A hesitação vacinal tem nuances, abrangendo desde aqueles que aceitam todas as vacinas sem dúvidas até aqueles que as recusam de forma inquestionável; os indivíduos hesitantes estão localizados entre esses dois extremos. O grupo também propôs um modelo de análise da hesitação vacinal que, inicialmente, remete a três determinantes: (1) confiança, que é o conhecimento e a percepção sobre segurança e eficácia das vacinas e do sistema que as fornece, incluindo os serviços e profissionais de saúde, e a motivação dos formuladores de políticas para recomendá-las; (2) conveniência, que envolve a disponibilidade, a acessibilidade geográfica dos serviços de vacinação, o acesso à informação e a capacidade de compreensão; e (3) complacência, que diz respeito à baixa percepção individual do risco e do valor atribuído às vacinas ^11^. Com o avanço dos estudos na literatura internacional, foram propostos mais dois elementos, sendo eles: (4) a comunicação, que diz respeito à infodemia, consubstanciada no excesso de informações; e (5) o contexto, que diz respeito a etnia, religião, ocupação e *status* socioeconômico como fatores estruturais que podem levar a uma baixa adesão à vacina em alguns grupos ^12^.

No Brasil, um estudo realizado na atenção primária à saúde (APS) do Estado do Maranhão aplicou a escala desenvolvida pelo SAGE e classificou 25,2% dos 246 responsáveis entrevistados com comportamento hesitante. A pesquisa aponta o medo de reações graves e a falta de conhecimento sobre a imunização como principais motivos da incerteza ^13^. Um estudo mais abrangente, realizado *online* em todos os estados brasileiros com 173.178 participantes em janeiro de 2021, demonstrou que 11,9% dos pais apresentaram hesitação vacinal. O medo da reação adversa aparece como principal causa da insegurança nesse grupo, mesmo entre aqueles que se declararam a favor das vacinas tradicionalmente aplicadas. Cerca de 7,7% dos entrevistados demonstraram hesitação vacinal para a vacinação de COVID-19, revelando preocupação específica com a segurança dessa vacina ^14^. Já uma pesquisa realizada em 23 países, com 1.000 participantes em cada, demonstrou que a hesitação vacinal infantil aumentou 56,3% no Brasil em 2021 ^15^.

Assim, considerando a descoordenação entre os entes federativos durante a pandemia da COVID-19, a desinformação e as ações do Governo Federal, que descredibilizaram os imunizantes infantis, este trabalho objetiva analisar a percepção da hesitação vacinal infantil da vacina contra a COVID-19 no Brasil, ponto no qual almeja ser inovador.

## Método

Este é um estudo qualitativo baseado no conceito de percepção social da psicologia social, entendida como uma construção social e histórica sobre um evento, pessoa ou situação. Essa é uma vertente construcionista da percepção dos sujeitos a partir da perspectiva da pessoalidade, envolvendo trocas simbólicas, negociação e especialmente interpessoalidade ^16^. O estudo foi realizado com 86 profissionais de saúde em quatro municípios brasileiros (Rio de Janeiro, Rondonópolis/Mato Grosso, Feira de Santana/Bahia e São Paulo) e no Distrito Federal. A escolha dos municípios não teve intenção amostral, mas contemplou capitais e cidades do interior com maior zona rural e de diferentes regiões brasileiras. A escolha por profissionais de saúde se justifica por serem considerados formadores confiáveis de opinião sobre a vacinação, podendo contribuir com a adesão ou com a hesitação vacinal ^17^.

O recrutamento e a coleta de dados ocorreram entre janeiro e dezembro de 2022. Foram realizadas entrevistas semiestruturadas presenciais e remotas com questionário dividido em quatro blocos temáticos, a saber: (1) perfil sociodemográfico; (2) experiência na vacinação contra COVID-19 e a hesitação vacinal; (3) desigualdade em saúde nos territórios; e (4) desafios da vacinação e experiências e opiniões sobre a vacinação contra a COVID-19. Todos os entrevistados consentiram em participar da pesquisa.

Para a seleção dos participantes, em um primeiro momento, foram entrevistados profissionais de saúde da APS, mediante escolha por conveniência das unidades de saúde das diferentes localidades estudadas. O estudo priorizou o recrutamento de profissionais que compõem a equipe mínima da Estratégia Saúde da Família, ou seja: médico, enfermeiro, auxiliar e/ou técnico de enfermagem e agente comunitário de saúde e/ou agente de endemias. No entanto, os convites se estenderam a profissionais de saúde, tais como dentistas, educadores físicos e farmacêuticos atuantes em Núcleos Ampliados de Saúde da Família e Atenção Básica (NASF-AB) ou nas equipes de saúde bucal.

O critério de inclusão era ser profissional de saúde e ter trabalhado por pelo menos seis meses entre 2020 e 2022. A inclusão de participantes de diferentes regiões possibilitou a diversidade das respostas. Neste estudo não se objetivou obter representatividade dos profissionais de saúde brasileiros.

Prosseguiu-se com a amostragem em bola de neve, com o intuito de identificar a produção de sentidos produzidos pelos profissionais de saúde por saturação. Uma vez esgotados/saturados os convites para recrutamento entre os profissionais de saúde das duas unidades elencadas, continuou-se com a amostragem para ampliar o alcance da pesquisa e capturar narrativas diversificadas sobre a percepção do fenômeno da hesitação vacinal.

A Tabela 1 apresenta o perfil sociodemográfico e a distribuição dos participantes segundo o município em estudo. Dos 86 participantes, 29 (33,7%) eram do Rio de Janeiro, 22 (25,6%) de Rondonópolis, 20 (23,3%) de Feira de Santana, 8 (9,3%) de São Paulo e 7 (8,1%) de Brasília; 70 (81,4%) eram do sexo feminino, maior parte (41,9%) tinham entre 37 e 56 anos e raça/cor predominantemente branca (39,5%).

**Tabela 1:** Perfil sociodemográfico e distribuição da população.

Características	Participantes (%)
Gênero	
Feminino	70 (81,4)
Masculino	16 (18,6)
Profissão	
Agentes comunitários de saúde	21 (24,4)
Enfermeiros	25 (29,1)
Médicos	16 (18,6)
Odontologistas	2 (2,3)
Técnicos de enfermagem	20 (23,3)
Outros	2 (2,3)
Idade (em anos)	
25-36	30 (34,9)
37-46	36 (41,9)
47-62	21 (24,4)
Raça/Cor	
Preto	16 (18,6)
Pardo	31 (36,0)
Branco	34 (39,5)
Amarelo	1 (1,2)
Dado faltante	4 (4,7)
Escolaridade	
Médio	15 (17,4)
Técnico	18 (20,9)
Superior	18 (20,9)
Pós-graduação	35 (40,7)
Campo de pesquisa	
Rio de Janeiro	29 (33,7)
Rondonópolis	22 (25,6)
Feira de Santana	20 (23,3)
São Paulo	8 (9,3)
Brasília	7 (8,1)

As profissões dos participantes incluíram agente comunitário de saúde - ACS (24,4%), enfermeiro (29,1%), médico (18,6%), odontologista (2,3%), técnico de enfermagem (23,3%) e outros (2,3%). Quanto à escolaridade, 17,4% tinham nível médio (15), 20,9% técnico (18), 20,9% superior (18) e 40,7% pós-graduação (35).

As entrevistas duraram cerca de 45 minutos, foram transcritas e, posteriormente, codificadas com o auxílio do software Dedoose (https://www.dedoose.com) para a realização da análise temática ^18^. Ao longo da leitura e da interpretação das narrativas, foram atribuídos códigos indutivos, que foram estabelecidos pela equipe de pesquisadores mediante extensa análise do material empírico e conforme o roteiro de entrevista, alinhado aos objetivos de estudo. Com o apoio dessa sistematização de dados, três temas destacaram-se acerca da hesitação na vacinação contra a COVID-19 em crianças, como demonstra o Quadro 1.

**Quadro 1:** Apresentação dos temas e seus respectivos códigos indutivos elaborados a partir da coleta de dados.

TEMAS	CÓDIGOS INDUTIVOS
Medo	A vacina ainda é experimental Reações adversas Ausência de estudos de longo prazo Falsa percepção de risco menor da COVID-19 em crianças Condutas do Governo Federal geradoras de insegurança nos efeitos da vacina
Desinformações sobre a vacina	*Fake news* sobre a vacina e suas reações Infodemia e desinformação Orientação e conhecimento sobre vacinas
Papel dos profissionais de saúde na vacinação	Relatos sobre a influência na aceitação da vacina Histórico de confiança em vacinas

O estudo foi aprovado pela Comissão de Ética em Pesquisa da Escola Nacional de Saúde Pública Sergio Arouca, Fundação Oswaldo Cruz (ENSP/Fiocruz; nº 5.430.488), e as entrevistas foram conduzidas apenas após a aprovação do Termo de Consentimento Livre e Esclarecido (TCLE). Nos trechos de fala selecionados, apresentados na seção *Resultados e Discussão*, optamos por identificar apenas o município da atuação profissional dos entrevistados para preservar o sigilo das entrevistas.

## Resultados e discussão

Para a sistematização e discussão dos resultados da pesquisa, os códigos indutivos foram agrupados em três grandes temas de análise e buscaram refletir a percepção da incerteza relacionada à vacina infantil de COVID-19 no Brasil. Os determinantes da hesitação vacinal (confiança, conveniência, complacência, comunicação e contexto), descritos frequentemente na literatura para organizar e definir a hesitação vacinal, foram utilizados como referencial teórico e emergem de maneira transversal aos códigos indutivos e aos temas deste estudo, conforme demonstrado a seguir.

### Medo

Após um início conturbado da imunização em idosos e adultos, a forma pela qual ocorreu a vacinação das doses pediátricas gerou maior insegurança. O cenário político e social, as opiniões divergentes sobre a importância da vacinação infantil e a grande circulação de informações falsas foram associados à palavra “medo” nas respostas dos participantes, principalmente quando questionados acerca dos motivos da hesitação vacinal infantil. O “medo” foi manifestado de diversas formas e invocado por diferentes razões.

Destacamos que a maioria dos profissionais de saúde que participaram deste estudo declararam compreender a vacinação infantil contra a COVID-19 como muito importante e vacinaram ou expressaram a intenção de vacinar seus filhos no período estipulado pelo calendário vacinal. No entanto, um número expressivo também relatou ter experimentado medo de vacinar seus filhos e/ou dependentes em um primeiro momento.

Além disso, os profissionais entrevistados perceberam diferenças entre a imunização do público adulto e infantil. Enquanto o primeiro foi marcado pelo grande fluxo diário, principalmente após a vacinação dos grupos prioritários e idosos, a vacinação infantil teve baixa procura, com dificuldade de atingir as metas para a imunidade coletiva e com desconfiança por parte dos responsáveis.

O “medo” pode ser interpretado como uma reação emocional perante a percepção de uma ameaça iminente, sendo irrelevante se a percepção é real ou exagerada. Logo, o medo resulta em uma reação defensiva que demonstra a identidade e a fragilidade de uma pessoa, cultura ou civilização ^19^.

Diante das milhares de mortes por COVID-19 e o desenvolvimento de vacinas eficazes para a doença, o medo, no lugar da esperança, aparenta ser um afeto contraditório. Apesar da imunização infantil ser uma das intervenções de saúde pública mais bem-sucedidas e econômicas no Brasil e no mundo ^20^, a associação da vacinação contra a COVID-19 com o perigo demonstra a necessidade de melhores reflexões sobre o fenômeno.

As declarações sobre o medo de vacinar as crianças, que podemos interpretar aqui levando-se em conta o determinante confiança do conceito de hesitação vacinal, estão associadas à rapidez com a qual os imunizantes foram desenvolvidos e à crença de que a vacina ainda é experimental devido à ausência de estudos de longo prazo. Além disso, também há o medo das reações adversas e questões sobre sua segurança.

“*...Outras pessoas não* [se] *vacinam por não saber de fato os efeitos da vacina a médio e longo prazo.* (...) *Por medo também de pegar, de tomar a vacina e ter a doença. E esses ruídos na comunicação acabam trazendo medo, trazendo alguma dúvida, pois a vacina do COVID é uma vacina nova...*” (Brasília).

A crença de que os imunizantes são experimentais aparenta ter grande influência pois não têm precedentes nas vivências dos usuários do sistema de saúde, gerando desconfiança e medo.

“*A gente explica que a vacina da COVID-19 é segura, que é uma vacina que realmente foi testada,* (...) *aí prefere fazer* [somente] *a outra, que é de rotina, aí a gente fala: ‘mas a vacina é a mesma praticamente, o mesmo local que são feitos, o mesmo teste que foi feito nessas vacinas foi feito na vacina de COVID’*” (Feira de Santana).

O medo das reações adversas também foi nomeado por meio do determinante da complacência, ou seja, a percepção de que os riscos dos efeitos da vacina são maiores do que a possibilidade de seus filhos desenvolverem a forma grave de COVID-19. Alguns profissionais entrevistados se mostraram complacentes com a necessidade de vacinar as crianças em razão da sua percepção individual de poucos ou nenhum caso grave nessa faixa etária.

A suposição de que as crianças são assintomáticas e/ou que têm imunidade natural foi defendida por autoridades do Governo Federal, como a ex-secretária de Gestão do Trabalho e da Educação na Saúde, Mayra Pinheiro, em maio de 2020, que afirmou que as medidas de isolamento social “*atrapalharam a evolução natural da doença naquelas pessoas assintomáticas, como as crianças*” ^21^ (p. 54), impedindo o efeito rebanho.

O relato dos participantes da vacinação associada ao perigo, gerando o medo, demonstra o desconhecimento sobre os riscos da COVID-19 à população infantil e a importância da vacinação dessa faixa etária para que se alcance a imunidade coletiva (e não como uma decisão individual ou familiar). De acordo com o Ministério da Saúde ^22^, até novembro de 2023 foram registrados 5.310 casos de síndrome respiratória aguda grave (SRAG) por COVID-19 e 135 óbitos de SRAG por COVID-19 entre crianças menores que cinco anos. Além disso, foram registrados 2.115 casos de síndrome inflamatória multissistêmica em crianças (SIM-P) no Brasil, com 142 óbitos entre crianças e adolescentes.

O medo também é citado quando se faz referência ao termo de responsabilidade assinado pelos pais e tutores para vacinar as crianças, exigido pelo Ministério da Saúde em um primeiro momento da vacinação infantil. De forma inédita, a diretriz do Governo Federal exigiu uma declaração por escrito dos pais ou tutores afirmando estarem de acordo com a vacinação contra a COVID-19 ^23^^,^^24^, uma ação que possivelmente contribuiu para a desconfiança no Programa Nacional de Imunização (PNI). De acordo com os participantes, isso foi percebido como uma isenção por parte do Estado, o qual deixou de garantir a segurança dos imunizantes disponibilizados à população, assim como uma responsabilização inadequada dos familiares.

A ação do Governo Federal também foi associada às falas de descrédito às vacinas do ex-presidente Jair Bolsonaro, o qual afirmou que sua filha não tomaria a vacina ^25^ e que o efeito das vacinas em crianças seria uma incógnita ^26^. Falas como essas, de acordo com os entrevistados, foram proferidas pelos usuários dos serviços, corroborando o medo e a insegurança que sentiram em relação ao imunizante. Não por acaso, alguns entrevistados citam a intervenção negativa do ex-presidente na adesão à vacinação infantil.

“*Só que são questões diferentes que impedem a adesão. Essa questão de acreditar que a vacina não funciona, acreditar que a vacina tá batizada, que a vacina vai controlar o cérebro, nessas questões ideológicas, funcionam mais intensamente com o COVID.* (...) *como é que eu vou te dizer? - o presidente disse que não é pra tomar vacina, né?*” (Rio de Janeiro).

A forma com que o “medo” foi mencionado pelos entrevistados causa reflexões sobre a condução da pandemia por parte do Governo Federal, principalmente no que tange à governabilidade por meio desse afeto e do uso das plataformas digitais como meio de acesso à informação pela população. De acordo com Letícia Cesarino ^27^ (p. 232), “*conteúdos heterodoxos têm maior probabilidade de gerar engajamento e compartilhamento: pela novidade, pelo exotismo, pelo caráter revelatório, por incitar afetos de medo ou indignação*”. Assim sendo, enquanto as reações vacinais e os efeitos adversos geram engajamento e compartilhamento, a rotina de uma sala de vacinação não tem o mesmo apelo.

Nesse cenário, o espectro político vinculado ao ex-presidente Jair Bolsonaro reforçou narrativas conspiratórias com relatos de efeitos adversos e mortes pós-vacina ^28^, enquanto pais e responsáveis com medo foram entregues à sua própria sorte em relação aos cuidados das crianças. Assim como em outros períodos da história, percebe-se que durante a pandemia da COVID-19, o medo se tornou um dos argumentos centrais da política ^29^, de forma que essa resposta emocional à percepção de um perigo ^19^ foi mobilizada e acentuada, com consequências para a hesitação vacinal infantil.

### Desinformação sobre a vacina

A forma e o conteúdo das informações sobre a vacina contra a COVID-19 em crianças nos ajudam a compreender vários aspectos dos determinantes comunicação e confiança da hesitação vacinal, ambos abalados pelas dificuldades colocadas pela infodemia e pela desinformação.

A infodemia é um fenômeno relacionado à grande disseminação de informação, tornando difícil a identificação de suas fontes. Na pandemia, esse fenômeno foi catalisado pelo processo de politização do tema, resultando no exagero ou na subestimação da doença ^30^ e interferindo na confiança e na aceitação à vacina contra a COVID-19. Além disso, parte considerável do conteúdo que circula nas plataformas digitais é caracterizado como desinformação, ou seja, é incompleto, impreciso e não tem fontes confiáveis. Por ser compartilhado rapidamente, principalmente nas redes sociais, e ter um elevado poder de alcance, representa um desafio para respostas e estratégias de combate ^30^.

“*Eu acho que o tá influenciando é essa desinformação, esse monte de fake news, e as pessoas estão tendo mais acesso a essas informações e também à desinformação, né?* (...) *eu acho que as pessoas têm que se empoderar mesmo, têm que estudar, mas eu acho que é muito difícil entender, tem estudos que nem eu entendo, que sou da área da saúde, né? Então não dá pra pessoa chegar: ‘ah, li, entendi tudo’*” (Rondonópolis).

A fala em destaque exemplifica dois elementos que estão muitas vezes relacionados: o excesso de informação, viabilizado quase instantaneamente pela internet e pelas redes sociais, e as mensagens que visam produzir desinformação e/ou informações inverídicas, como as *fake news*.

As *fake news* foram percebidas pelos profissionais como uma das principais influências para a hesitação na vacinação de COVID-19 em crianças. Um inquérito *online*, realizado com 173.178 respondentes, investigou a confiabilidade das fontes de informação sobre a vacinação. Nessa pesquisa, houve a predominância dos não hesitantes em relação a vacinas, os quais relataram menor confiança nas informações do Ministério da Saúde uma vez que foram associadas à rotatividade de ministros e à falta de transparência na divulgação dos dados da pandemia. Já no grupo dos hesitantes, as fontes mais confiáveis foram os artigos científicos, seguidos por institutos de pesquisa e os médicos. Para esse grupo, a televisão e os jornais foram as fontes de informação menos confiáveis ^31^.

Os profissionais entrevistados citaram os membros da própria equipe de saúde como principais fontes de informação sobre a vacinação de COVID-19. Médicos e enfermeiros foram indicados como os profissionais de maior confiabilidade nesse sentido. A internet também foi mencionada, sendo os sítios eletrônicos das sociedades e conselhos de classe (como a Sociedade Brasileira de Pediatria e os Conselhos de Enfermagem e de Medicina), de jornais, da OMS, da Fiocruz, do Instituto Butantan e do Ministério da Saúde relatados como os mais confiáveis.

Sabe-se da importância do determinante da confiança na hesitação vacinal, tendo em vista que indivíduos com maiores níveis de confiança no governo e nas instituições de saúde e que os utilizam como fonte de informações são mais propensos a se vacinar contra o COVID-19. A confiança nas fontes das instituições de saúde e nas políticas governamentais podem contribuir não apenas para comportamentos individuais de aceitação da vacina, mas também para encorajar amigos e familiares na tomada de decisão ^32^.

Em nosso estudo, no entanto, a percepção de discursos divergentes entre os entes federativos da gestão do Sistema Único de Saúde (SUS) culminou em maior incerteza entre os pais e responsáveis, observada pela baixa adesão à vacina nos municípios. Diante dessa desarticulação, as secretarias municipais foram elencadas pelos profissionais como referências e fontes seguras.

“*...eu acho que eu confiaria mais no estado e nos municípios, sem dúvida. Eu acho que a atuação foi muito mais segura, mais pé no chão, do que a atuação do Governo Federal foi nessa pandemia. Eu não tenho a menor dúvida*” (Rondonópolis).

As divergências e as pressões políticas de parlamentares da base de sustentação do antigo governo se refletem na dificuldade de comunicar à população os riscos, sanar as preocupações referentes à vacina e divulgar a campanha de vacinação pelo PNI. Para reverter o cenário, ações estratégicas de divulgação de dados e de informações normativas, apoio de pessoas públicas e influenciadores nas mídias sociais podem motivar à população a maior aceitação da vacina ^33^. No entanto, nosso estudo corrobora com a premissa de que é necessário compreender o fenômeno da infodemia e desinformação em torno da temática para a criação de estratégias de comunicação mais eficazes.

### Papel dos profissionais de saúde na vacinação

No Brasil, a APS é uma das esferas nas quais o PNI é implementado e seus profissionais têm papel estratégico na execução do plano de imunizações e nas diversas tarefas inerentes a ele, para além da recomendação e aplicação das vacinas. A APS atua em ações de educação e comunicação que devem ser humanizadas e integrais, essenciais para a promoção, prevenção e o controle de doenças imunopreveníveis ^17^. Dado esse papel central, conhecer as experiências e entender a percepção dos profissionais de saúde é de grande utilidade para compreender a hesitação vacinal, principalmente no determinante da confiança, que engloba a confiabilidade e competência do sistema de saúde, dos formuladores de políticas e profissionais de saúde.

Observamos neste estudo que profissionais da APS de todos os campos em estudo nomearam o “medo” e as “*fake news*” como fatores preocupantes que fizeram com que as pessoas não se vacinassem ou hesitassem de alguma forma, principalmente em relação à vacinação infantil. Nesse cenário, esses mesmos profissionais utilizaram conversas, visitas às residências, comentários e respostas às dúvidas como uma forma de fazer a população aderir à vacina.

“*Eu tive várias conversas durante a campanha sobre vacinação, me colocava à disposição para tirar dúvidas sobre vacinação, e tinha pacientes que tiravam dúvidas e a gente encontrava gente convicta de não vacinar também*” (Brasília).

Dessa forma, os profissionais consideraram que fornecer informações sobre a eficácia e a segurança da vacina contra a COVID-19 aos pais/responsáveis influenciou positivamente na aceitação da vacinação infantil. Isso se dá, principalmente, quando se dispõem a sanar dúvidas sobre as possíveis reações adversas das vacinas, motivo recorrente de insegurança segundo os entrevistados.

“*A gente explica que é uma vacina segura, que é uma vacina que realmente foi testada, e mostra a questão do manual que é direcionado para a gente, tem as informações onde são testadas, como é testada essa vacina, como ela chega até a unidade de saúde*” (Feira de Santana).

De acordo com os entrevistados, os pais/tutores hesitantes atribuem aos profissionais maior capacitação e conhecimento em saúde, inspirando confiança na população ^34^. Nesse contexto, os profissionais médicos, por serem validados socialmente como atores centrais no cuidado dos pacientes, têm uma grande capacidade de influenciar na decisão dos usuários dos serviços de saúde ^35^. Portanto, assim como os profissionais, sobretudo os médicos, são sujeitos capazes de influenciar e gerar confiança, também podem causar incerteza quando adotam posturas contrárias às vacinas, tendo em vista a posição de poder que ocupam.

“*Você vê que tem médicos que chegam nas televisões e falam que a vacina não presta, que isso, que aquilo - que era pra estarem apoiando, né? Porque a nossa segurança vem do médico, digamos assim, o médico é estudado para isso, ele estuda, então ele conhece. Então se o médico tá dizendo que é bom, eu vou acreditar que é bom. A partir do momento que entra um médico, e diz que não é bom, aí confunde a cabeça da pessoa, a pessoa fica insegura, porque quem era para dizer ‘tome a vacina, está tudo OK’, que é bom, não está fazendo isso*” (Brasília).

Os profissionais da enfermagem e técnicos de enfermagem ganham destaque nas estratégias adotadas para proporcionar maior segurança aos pais com relação à vacinação contra a COVID-19 durante o processo de acolhimento, nas visitas domiciliares, durante os procedimentos na unidade ou na própria sala de vacina.

“*E aí, assim, a gente explica a importância da vacinação, mostra a quantidade de crianças que, né, já foram salvas em UTI* [unidade de terapia intensiva]*, né, de internação por conta... E, assim, tenta encorajar, né, pai e mãe a vacinar*” (São Paulo).

O trabalho dos ACS tornou-se igualmente estratégico e relevante no processo de vacinação das crianças, considerando que estão inseridos no território e realizaram busca ativa das crianças não vacinadas. Nesse sentido, a atuação dos ACS permite a aproximação e o diálogo com os usuários, assim como possibilita o estabelecimento de relações de confiança para uma abordagem que os incentive à vacinação.

“*Nós, como agente de saúde, temos um certo vínculo com o paciente, eu acho que cada profissional de saúde, não só o agente de saúde, como o médico da área, ou o enfermeiro, tentamos buscar informar essa pessoa, eu acho que seria um jeito pra essa pessoa se orientar mais*” (Rio de Janeiro).

Percebe-se que, enquanto a população sentia medo da vacinação, associado à grande produção e circulação de desinformação, a conversa gerada pela APS foi determinante na adesão aos imunizantes, especialmente a infantil. Esse dado é de extrema relevância quando pensamos na disputa de discursos acerca da vacinação, principalmente nos espaços virtuais.

## Considerações finais

A pandemia de COVID-19 ocasionou diversos desafios para os sistemas nacionais de saúde, entre eles: a adesão vacinal em contextos de incertezas em relação a uma nova enfermidade; as dúvidas advindas das vacinas produzidas em tempo recorde e distribuídas inicialmente em países desenvolvidos; e o aprofundamento das adversidades políticas e econômicas em uma situação de instabilidade. Nesse sentido, este trabalho apresentou uma análise da percepção dos profissionais de saúde da APS sobre a vacinação infantil contra a COVID-19.

Esta análise buscou ir além dos problemas de disponibilidade do imunizante e da descrição e categorização dos motivos de insegurança listados pelos participantes ao discutir as interfaces das motivações que resultam em uma postura de não adesão. Assim, quando os profissionais da saúde mencionam o medo como um dos motivos pelos quais as pessoas deixam de se vacinar, mesmo tendo acesso aos imunizantes, estão abordando alguns determinantes da hesitação: a complacência, a confiança e a desinformação. Além disso, o tema também abrange os afetos da população, que se percebeu em perigo iminente perante os imunizantes.

Já no que tange à desinformação, os profissionais relataram que a disseminação de informação afetou a assimilação (ou não) de quais medidas seriam adequadas por parte da população, gerando dúvidas acerca da vacinação. Contra a desinformação, os profissionais pontuaram a necessidade de enfrentar as *fake news* sobre as vacinas por meio de políticas públicas que disponibilizem informações claras e precisas sobre os imunizantes, de acordo com as particularidades dos seus territórios.

Pela primeira vez na história do PNI, órgãos públicos de comunicação foram responsáveis pela disseminação de desinformação e desincentivo a uma vacina, o que se relaciona com a ausência de coordenação federal sobre a vacinação, fatos determinantes para a hesitação vacinal no Brasil. As disputas discursivas construídas em torno da COVID-19 e as falas que minimizaram a enfermidade, caracterizando-a como uma “gripezinha” ou como uma doença preocupante apenas para os grupos de risco, ajudaram a menosprezar a importância da imunidade coletiva pela vacina. Nesse cenário, esta pesquisa destaca a necessidade de instituições científicas e formuladores de políticas públicas retomarem a pauta da vacinação no intuito de ser objetiva na comunicação em saúde, e não meramente responsiva à desinformação.

Vale ressaltar que o escopo do trabalho não explorou outros condicionantes da hesitação vacinal, como as estratégias de comunicação pública sobre as vacinas contra a COVID-19, a atuação de estados e municípios para promover o acesso e a adesão às vacinas, a formação e o conhecimento dos profissionais sobre o campo das vacinas, entre outros, o que representa uma limitação.

Este artigo divulga parcialmente os resultados alcançados na pesquisa. A hesitação vacinal é um novo campo de estudo no Brasil, especialmente em relação às crianças, demandando novas pesquisas que abordem a percepção dos responsáveis em desenhos que respondam aos desafios sociais, políticos e culturais característicos da diversidade brasileira.

Por fim, as percepções acerca do processo da imunização aqui compartilhadas somente se tornaram acessíveis a partir da proximidade que os entrevistados estabeleceram com o seu território, utilizando a vivência de seus afazeres para a compreensão da conjuntura imposta. Dessa forma, os profissionais de saúde da APS se mostraram atores-chaves para a reconstrução e/ou manutenção da confiança na vacinação, pela estrutura do modelo de atenção, pelo estabelecimento de vínculo com a população e o território e pela disponibilização de informações em saúde, contribuindo com as políticas de vacinação, principalmente na população infantil.

Nesse sentido, o fortalecimento e valorização desses trabalhadores demandam condições de trabalho e salários dignos, formação adequada e permanente, além de apoio governamental nas estratégias de comunicação coletiva à sociedade.
